# Investigating Resistance to Carbapenems in Enterobacterales: A Descriptive Epidemiological Study of 2021 Screening in an Italian Teaching Hospital

**DOI:** 10.3390/pathogens12091140

**Published:** 2023-09-06

**Authors:** Erica De Vita, Luigi De Angelis, Guglielmo Arzilli, Francesco Baglivo, Simona Barnini, Alessandra Vecchione, Angelo Baggiani, Caterina Rizzo, Andrea Davide Porretta, Teams AID

**Affiliations:** 1 Department of Translational Research and New Technologies in Medicine and Surgery, University of Pisa, 56123 Pisa, Italy; erica.devita@unipi.it (E.D.V.); l.deangelis2@studenti.unipi.it (L.D.A.); f.baglivo@studenti.unipi.it (F.B.); angelo.baggiani@unipi.it (A.B.); caterina.rizzo@unipi.it (C.R.); andrea.porretta@unipi.it (A.D.P.); 2 Microbiology Unit, University Hospital of Pisa, 56124 Pisa, Italy; s.barnini@ao-pisa.toscana.it (S.B.); a.vecchione@ao-pisa.toscana.it (A.V.); 3 University Hospital of Pisa, 56123 Pisa, Italy

**Keywords:** antimicrobial resistance (AMR), enterobacterales, rectal swabs, hospital screening, carbapenem-resistant enterobacterales (CRE), infection control, multidrug-resistant organisms (MDROs), carbapenemase-producing enterobacterales (CPE), New Delhi metallo-beta-lactamase (NDM)

## Abstract

Antimicrobial resistance (AMR) presents a growing threat to global healthcare. This descriptive epidemiological study investigates the prevalence and characteristics of Enterobacterales with AMR factors in a tertiary teaching hospital in Italy over the course of the year 2021. In 2021, the prevalence of colonisation by Enterobacterales with AMR factors in patients was 1.08%. During the observation period, a total of 8834 rectal swabs were performed, with 1453 testing positive. A total of 5639 rectal swabs were performed according to a hospital procedure for the active screening of MDRO colonisation at the time of admission. Of these, 679 were positive for microorganisms under surveillance, and 74 patients were colonised with Enterobacterales, predominantly *Klebsiella pneumoniae* and *Escherichia coli*. Antibiotic resistance factors were observed in 61 of these 74 patients (82.43%) of these patients, with NDM and KPC being the most frequent resistance factors. A statistically significant trend in positive swabs was observed across different ward categories (surgery, ICUs, and medical wards). Regarding specific trends, the rate of positive admission screening in medical and surgical wards was higher than in ICU wards. The results highlight the ease with which Enterobacterales develops resistance across different ward categories. The findings underscore the need for adjusted screening protocols and tailored infection prevention strategies in various care settings.

## 1. Introduction

The increasing emergence in the hospital environment of multidrug-resistant organisms (MDROs), including resistance to last-resort drugs, such as carbapenems, is of particular concern, as it makes it challenging to treat patients effectively, and is increasingly being reported worldwide [1]. Infections sustained by these microorganisms contribute to the burden of healthcare-associated infections (HAIs) on longer hospitalisation, higher healthcare costs, and increased mortality (702.53 DALYs per 100,000 general population) [2,3,4,5,6].

Enterobacterales, a large family of Gram-negative bacteria, including well-known pathogens such as *Escherichia coli* and *Klebsiella pneumoniae*, poses a significant challenge in infection control, due to its capacity to accumulate multiple resistance mechanisms [7].

The growing resistance to carbapenems has caused several outbreaks of nosocomial infections worldwide, with them becoming endemic in several countries across Europe, Asia, South America, and Africa [8]. In Europe, resistance to carbapenems has been reported in most countries, albeit with different prevalence. The epidemiological diversity extends beyond prevalence, and includes variations in the types of prevalent carbapenemases among carbapenemase-producing Enterobacterales (CPEs), as evidenced by the EuSCAPE survey [9]. The recent emergence of specific resistance mechanisms, such as New Delhi metallo-beta-lactamase (NDM), *Klebsiella pneumoniae* carbapenemase (KPC), oxacillinase-48 with carbapenemase activity (OXA-48), and Verona integron-encoded metallo-beta-lactamase (VIM), has further complicated the situation of multidrug resistance in Enterobacterales [10].

Italy was among the first countries in Europe to report CPE isolation, and experienced rapid and extensive dissemination of CRE, primarily driven by *Klebsiella pneumoniae* strains producing KPC-type carbapenemases (KPC-Kp) [11]. The epidemiological background in Italy differs from that in other highly endemic European countries, such as Greece, where VIM-producing strains predominated initially, and were later compounded by the presence of KPC-Kp and OXA-48 producers.

In Italy, the CRE epidemic has expanded nationwide, and is now observed not only in inpatients from acute-care hospitals, but also among outpatients and patients in long-term care facilities. The reported prevalence of CRE carriage varies across settings, reflecting differences in patient populations, screening strategies, and study periods [12,13].

MDRO colonisation is a prerequisite for infections caused by the same pathogens [14]. According to the results of a 2016 meta-analysis, patients colonised with CRE have an overall 16.5% risk of infection [15]. However, given the high mortality rate observed with CRE infections (especially bloodstream infections [16,17]), and the difficulty in treating them, it is critical to prevent colonisation, by implementing patient isolation and contact precautions when needed, to avoid patient-to-patient transmission.

A program of early screening measures to identify patients carriers of CRE has been proposed, among other measures to control the diffusion of these MDROs [12]. It has been shown that failures in the early identification of CRE carriers have facilitated patient-to-patient transmission, contributing to the rapid escalation of outbreaks [18]. Resource constraints and the lack of consensus on optimal approaches have hampered the wider adoption of more proactive surveillance policies that could effectively reduce the prevalence of CRE. The lack of worldwide standardised protocols, and a non-uniform application of preventive measures, have limited the effectiveness in containing outbreaks and preventing HAIs. Crucial elements of surveillance programs, including the target population to be screened, the testing frequency, and the preferred diagnostic methods, continue to be debated [19].

Among the various methods available for detecting CRE carriage, rectal swab screening has emerged as a widely adopted approach, due to its practicality, cost-effectiveness, and capability of identifying CRE colonisation in the gastrointestinal tract, particularly in patients with conditions associated with multiple exposures to healthcare settings and antimicrobial agents [20]. Thus, rectal swab screening allows for the detection of CRE carriage in asymptomatic individuals, enabling early identification, and the subsequent implementation of infection control measures, to prevent transmission.

Despite the widespread utilisation of rectal swab screening, challenges remain regarding the optimal frequency of testing, and the interpretation of results. Some institutions advocate for universal screening upon admission, while others propose targeted screening based on risk factors such as recent healthcare exposure or previous colonisation [21]. Furthermore, the role of serial screening to detect new acquisitions or persistent carriage of CRE necessitates further investigation.

This study aims to describe the epidemiology of NDM, KPC, ESBL, OXA-48, and VIM factors in Enterobacterales detected via the screening procedure in a population of patients admitted to a large teaching hospital in Italy, an endemic area for these resistance patterns.

## 2. Materials and Methods

### 2.1. Data Source

We conducted an epidemiological descriptive analysis of microbiological laboratory data, using samples obtained from all patients admitted to the tertiary teaching hospital in Pisa (Tuscany, Italy) between 1 January 2021 and 31 December 2021, in accordance with the regional standard procedure for screening on admission, aimed at the early identification of CPE/CRE carriers, published in July 2019 [22]. In 2020, our hospital put in place a task force to face the COVID-19 pandemic that developed a technical procedure, structured in five key domains: the reorganization of hospital services, the management of suspected or confirmed COVID-19 patients, the management of corpses, guidelines for cleaning and disinfection, the implementation of cleaning and disinfection procedures, and personal protective equipment [23]. The rationale for choosing the study period has a contextual significance. After the release of the abovementioned guidelines, 2021 emerged as the pivotal focal point of our investigation. If, in 2020, non-emergency hospitalizations were minimized due to the early stages of the COVID-19 pandemic, in 2021, a rise in non-emergency hospitalizations, despite the pandemic still being ongoing, was seen.

The hospital procedure required testing with a rectal swab for active screening of MDRO colonisation at the time of admission (within 24 h) in patients with a previous history of access to the health system in the previous 12 months, or admitted to high-risk wards, such as haematology, intensive care units (ICUs), transplant surgery, heart surgery, the infectious diseases ward, the medical area, and rehabilitation wards. In the case of negative results, the swab was repeated every 7 days until discharge.

For the purpose of this study, we extracted the pathogen data anonymously, from the records of the laboratory database. The resulting dataset included the date of birth of patients, the ward of admission, the date of admission, the date of the test, the type of sample and, if a positive result was obtained, the organism identified and its phenotype of antimicrobial resistance. We grouped the patient data via their nosocomial identification codes, to avoid any double-counting of laboratory records. Only the first positive record for each patient was retained for further analysis, including for those with multiple admissions.

We compared the date of sample acceptance with the hospital admission date, to distinguish patients with pre-admission positivity from those who acquired in-hospital colonisation. We defined an admission screening test as a rectal swab with a difference of less than two days between sample acceptance and hospital admission.

For the study analyses, we selected a subset of the database, including only rectal swabs with a positive result for Enterobacterales, namely: *Enterobacter cloacae*, *Escherichia coli*, *Klebsiella* spp. (in particular, *Klebsiella pneumoniae* and *Klebsiella oxytoca*), and *Enterobacter cloacae complex*. We considered NDM, KPC, ESBL, OXA-48, and VIM as AMR factors for the target germs. For each patient with a rectal colonisation, the presence of a bloodstream infection (BSI) caused by the same pathogen was assessed.

We grouped all admission wards into specific categories: (1) medical wards, including general medicine wards and medical specialty wards (endocrinology, cardiology, gastroenterology, the infectious diseases ward, nephrology, pneumology, neurology, oncoheamatology, paediatrics, the accident and emergency ward, rheumatology, and immunology); (2) surgical wards, including general surgery wards, surgical specialties (urology, cardiothoracic surgery, vascular surgery, neurosurgery, ophthalmology, orthopaedics, and gynaecology), and transplant units; and (3) ICUs, including general ICUs, post-surgical ICUs, the COVID-19 ICU, and the burn ICU.

For the statistical analysis, we stratified our samples into four subgroups, contained within each other: (1) all positive rectal swabs for microorganisms under surveillance; (2) positive admission screening tests for any microorganism under infection control surveillance; (3) positive admission screening tests for Enterobacterales; and (4) positive admission screening tests for Enterobacterales with at least one AMR factor.

### 2.2. Statistical Analysis

A descriptive analysis of the data was performed. We reported AMR factors collected from rectal swabs according to the type of identified bacteria. We stratified the four subgroups of the sample population according to age and ward categories. We applied the Test for Trend in Proportions to explore the stratification of the cases across the different sub-groups of rectal swab and ward categories. This test examines whether there is a significant trend in a proportion across sub-groups of positive rectal swabs. The independent variable was the type of microorganism identified from the rectal swab, ordered from the less specific to more specific criteria. The dependent variable was the proportion of cases in each ward category (medical wards, surgical wards, ICUs). All the analyses were performed using R software.

### 2.3. Microbiological Methods

The identification of bacteria was carried out using matrix-assisted-laser-desorption-ionization–time-of-flight mass spectrometry (MALDI Biotyper, Bruker Daltonics). Antimicrobial susceptibility testing was performed using Phoenix, and MERLIN Diagnostika panels. Rectal swabs were plated onto ChromID Carba Smart plates (bioMérieux). The RESIST-3 O.K.N. immunochromatographic assay (Coris BioConcept), or a molecular test, was performed to establish the type of bacterial resistance. The molecular characterization of carbapenemase genes was conducted on the total amount of positive swabs, using either the Xpert CarbaR real-time PCR test (Cepheid) or the Allplex Entero-DR Assay (Seegene).

### 2.4. Ethical Considerations

The health data presented in this paper were collected from the routine surveillance system, and were anonymised before analysis, in compliance with the Declaration of Helsinki [24] and Italian regulations [25].

## 3. Results

**Figure 1:**
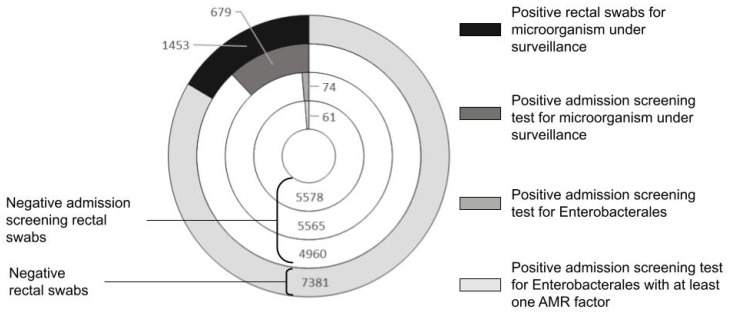
Screening of hospital inpatients for detecting rectal carriers for microorganisms under surveillance (Pisa Teaching Hospital, from January to December 2021).

From 1 January to 31 December 2021, the tertiary teaching hospital of Pisa performed 8834 rectal swabs (1453 were positive; 16.44%), of which 5639 (63%) were admission screening tests. Among the latter, 679 tested positive, accounting for 12.04% of the total admission screening tests (Figure 1).

Among the positive admission screening swabs, 87 tests detected Enterobacterales: 73 were *Klebsiella pneumoniae*, 10 were *Escherichia coli*, and 4 were other species. Of the 87 admission screening samples, 13 were excluded as they were multiple positive tests from the same patient during the observational period; these patients were screened and showed positive results in different hospitalizations during the year. Thus, the total number of colonised patients with Enterobacterales was 74 (38 males).

Out of 74 patients, 61 (82.43%) were found to be colonised by antibiotic-resistant bacteria carrying at least one antibiotic resistance factor, accounting for 1.08% of all rectal swabs collected during the admission screening.

The most frequent antibiotic resistance factor was NDM, followed by KPC, as reported in Table 1. Three patients presented germs with multiple AMR factors.

Analysing the different sub-groups of swabs according to the age of the colonised patients, antibiotic-resistant Enterobacterales were found in the older ones, with a median age of 70.6 years, and a mean of 68.9 ± 16.2, as reported in Table 2.

The analysis of rectal swabs across ward categories revealed different rates of colonisation. The medical wards showed the highest number of positive cases (*n* = 638), followed by the surgical wards (*n* = 383) and the ICUs (*n* = 395). A wide proportion of screened rectal swabs positive for Enterobacterales exhibited antibiotic resistance factors, with the medical wards (5.7% (37/649)) showing the highest number of such cases, followed by surgical wards (4.2% (17/403)), and ICUs (1.7% (7/401)) (Table 3).

Analysing the number of positive rectal swabs for all the subgroups across the different quarters of 2021, we can observe that the number of all positive rectal swabs and of admission screening tests in the third and fourth quarters largely increased, as shown in Table 4.

This is not matched by an increase in the number of positive admission screening tests for Enterobacterales, or for Enterobacterales with at least one AMR factor.

Blood cultures were performed on all the colonised patients (*n* = 74). We observed that 11 (14.86%) patients had a BSI via the same colonising pathogen. In all cases, the identified infections were caused by *Klebsiella pneumoniae*; all patients except one were infected by strains with at least one AMR factor (6 NDM, 3 KPC, 1 VIM).

**Figure 2:**
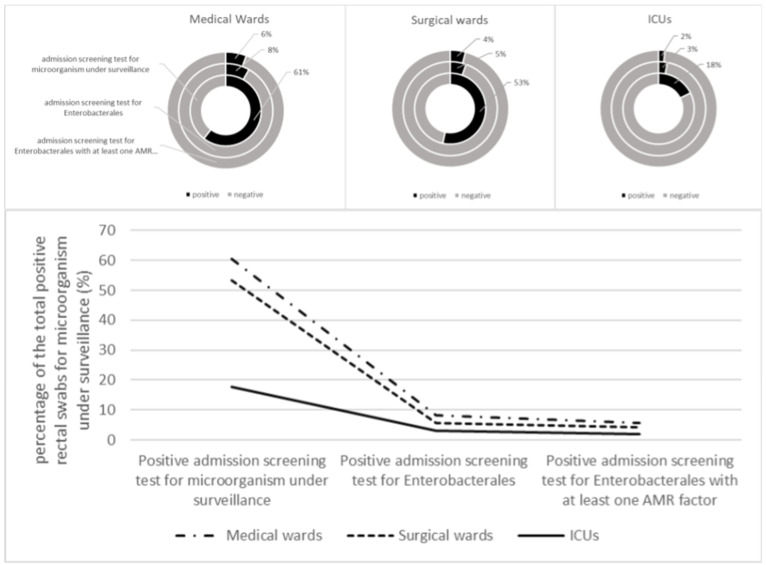
Upper panel: proportion of microorganisms stratified for different types of colonisations for each ward category—pie chart visualisation; lower panel: trend in proportion across the different types of colonisations for each ward category.

The Test for Trend in Proportions revealed a statistically significant trend (*p*-value < 0.001) in positive swabs across the different types of colonisations for each ward category (Figure 2).

In particular, we observed how the specific trends decreased from all positive rectal swabs, to Enterobacterales with at least one AMR factor, in all the types of wards.

## 4. Discussion

The effectiveness of active screening for detecting pathogens with AMR factors in patients admitted to hospital in everyday practice is still not well established [12].

In literature, the reported prevalence of these germs in rectal swabs was variable in different settings, reflecting a diverse patient case mix, screening strategies, and study periods [26,27,28,29]. In our study, the prevalence among the 5639 patients screened on admission for Enterobacterales with AMR factors was 1.08%. Such a prevalence aligns with data from previous studies: a prevalence ranging from 0.2 to 3.9% was reported among inpatients from acute care hospitals in northern Italy at the beginning of the CRE epidemic [30,31]. Nevertheless, the fragmentation of the observational studies on the assessment of Enterobacterales with AMR factors in carriers, and the setting-specific procedures applied, could mask the prevalence of the carriage of Enterobacterales with AMR factors, making it difficult to benchmark our data with other up-to-date data.

The most frequent AMR factor assessed in our study was NDM, both in *Klebsiella pneumoniae* and *Escherichia coli*, consistent with the large outbreak of NDM-CRE that occurred in Tuscany in 2018–2019 [32]. This outbreak changed the local epidemiology of CRE, despite the previous endemicity of KPC-producing CRE in this geographic area [33]. In our study, the resistance factor KPC closely follows NDM, consistently with the endemic diffusion of KPC-producing CRE in Tuscany.

Analysing the different sub-groups of positive rectal swabs, we can see that antibiotic-resistant Enterobacterales affected older patients, with a median age of 70.6 years, and a mean of 68.9 ± 16.2. This finding could be explained by older people experiencing more frequent exposure to healthcare assistance, and comorbidities that promote a more frequent use of antimicrobial drugs, including carbapenems [34].

Regarding specific trends, we observed that the rate of positive admission screening in medical and surgical wards was higher than in ICU wards. This trend was visualised in a line plot, clearly representing the changes in proportions across sub-groups of positive rectal swabs and ward categories. The clinical significance of these findings would require further investigation; however, we can assume that the longer length of stay in ICUs, work overload, more frequent invasive procedures, and extensive use of antimicrobial drugs may promote patient colonisation during hospitalization [35,36,37,38]. The different case mix of patients across ward categories could explain the outstanding difference in positive admission screening swab rates. While patients in clinical wards are more likely to have had previous contact with healthcare facilities and, therefore, are more likely to become carriers, it is crucial to implement infection prevention strategies in medical wards, surgical wards, and ICUs in endemic settings (such as Italy) and epidemic settings [39].

This study has several limitations. The study was conducted during the pandemic period (between 1 January 2021 and 31 December 2021) that impacted several aspects of healthcare assistance, particularly in hospital settings. Consequently, the available data for our study might have been affected: the pandemic-related restrictions, with the reduction of non-emergency hospital activities, may have led to a potentially limited and biased sample size. Further data collection and analysis are needed to better understand this phenomenon. In addition, the lack of clinical correlates to laboratory data, and the total amount of swabs performed by ward categories, meant that more detailed information on colonised patients could not be obtained, and the relative prevalence could not be calculated.

## 5. Conclusions

In light of the widespread global concern over antimicrobial-resistant bacteria in all healthcare settings, and the alarming prevalence of CRE/CPE in Italy, swift and comprehensive measures must be implemented, nationally and locally. This epidemiological study of resistance to carbapenems in a Tuscany teaching hospital provides insight into the healthcare setting in which the study takes place, highlighting the differences and similarities among different ward types. Thus, feeding the body of evidence may help to tackle the spread of resistance to carbapenems, by tailoring screening protocols to specific care settings and patient characteristics, e.g., age. Moreover, our study highlights that the epidemiological framework is still far from being solved and is similar to the prevalence found in the literature on MDROs.

## Figures and Tables

**Table 1:** Number of AMR factors distributed by bacterial species identified via the admission screening test.

	*Escherichia* *coli*	*Klebsiella pneumoniae* *	*Klebsiella oxycota*	*Enterobacter* *cloacae*
Total Number of Patients	9	61	1	3
NDM	3	26	//	//
KPC	2	23	//	//
ESBL	1	1	//	//
OXA-48	2	2	//	//
VIM	//	6	1	2
NO RES	1	6	//	1

* Three patients presented more than one antibiotic resistance factor. // = not detected

**Table 2:** Sub-groups of positive rectal swabs, stratified according to the age of the colonised patient (median, IQR, mean, standard deviation).

Sub-Groups of Positive Rectal Swabs	Age (Years)			
	Median	First Quartile	Third Quartile	Mean ± SD
All positive rectal swabs for microorganisms under surveillance	68.6	54.0	78.4	60.9 ± 25.1
Positive admission screening test for microorganisms under surveillance	70.0	55.6	79.9	63.3 ± 23.8
Positive admission screening test for Enterobacterales	70.4	58.6	79.8	66.4 ± 20.3
Positive admission screening test for Enterobacterales with at least one AMR factor	70.6	59.8	82.3	68.9 ± 16.2

**Table 3:** Sub-groups of positive rectal swabs stratified according to the ward category (medical wards, surgical wards, ICUs).

Sub-Groups of Positive Rectal Swabs	Identified in:		
	Medical Wards	Surgical Wards	ICUs
All positive rectal swabs for microorganisms under surveillance (*n* = 1453)	649	403	401
Positive admission screening test for microorganisms under surveillance (*n* = 679)	393	215	71
Positive admission screening test for Enterobacterales (*n* = 87)	53	22	12
Positive admission screening test for Enterobacterales with at least one AMR antibiotic resistance factor (*n* = 61)	37	17	7

**Table 4:** Number of positive rectal swabs stratified according to the time of hospital admission for each subgroup (first quarter: Jan–Feb–Mar; second quarter: Apr–May–Jun; third quarter: Jul–Aug–Sep; fourth quarter: Oct–Nov–Dec). The percentages are calculated using all positive rectal swabs for microorganisms under surveillance as the denominator.

	First Quarter of 2021	Second Quarter of 2021	Third Quarter of 2021	Fourth Quarter of 2021
All positive rectal swabs for microorganisms under surveillance	229	294	444	486
Positive admission screening test for microorganisms under surveillance	53 (23.1%)	88 (29.9%)	244 (55.0%)	294 (60.5%)
Positive admission screening test for Enterobacterales	17 (7.4%)	23 (7.8%)	19 (4.28%)	15 (3.1%)
Positive admission screening test for Enterobacterales with at least one AMR factor	13 (5.6%)	19 (6.5%)	16 (3.6%)	13 (2.7%)

## Data Availability

The data presented in this study are available on request from the corresponding author. The data are not publicly available due to privacy restriction and ethical issue.
